# Steady-state detection of evaporation process based on multivariate data fusion

**DOI:** 10.1371/journal.pone.0309652

**Published:** 2024-09-06

**Authors:** Xiaoshan Qian, Lisha Xu, Xingli Cui

**Affiliations:** 1 College of Physical Science and Engineering Technology, Yichun University, Yichun, Jiangxi, China; 2 College of Information Science and Engineering, Hunan Women’s University, Changsha, Hunan, China; GH Raisoni College of Engineering and Management Pune, INDIA

## Abstract

In this paper, we introduce an innovative multivariable data fusion strategy for adaptive steady-state detection, specifically tailored for the alumina evaporation process. This approach is designed to counteract the production instabilities that often arise from frequent alterations in production conditions. At the core of our strategy is the application of an adaptive denoising algorithm based on the Gaussian filter, which adeptly eliminates erroneous data from selected variables without compromising the fidelity of the original signal. Subsequently, we implement a multivariable R-test methodology, integrated with the adaptive Gaussian filter, to conduct a thorough and precise steady-state detection via data fusion. The efficiency of this method is rigorously validated using actual data from industrial processes.Our findings reveal that this strategy markedly enhances the stability and efficiency (by 10%) of the alumina evaporation process, thereby offering a substantial contribution to the field. Moreover, the versatility of this approach suggests its potential applicability in a wide range of industrial settings, where similar production challenges prevail. This study not only advances the domain of process control but also underscores the significance of adaptive strategies in managing complex, variable-driven industrial operations.

## 1 Introduction

In the Bayer wet process for producing alumina, the raw liquid (caustic soda solution) is concentrated using high-temperature and high-pressure fresh steam through evaporators, preheaters, flash evaporators, and other equipment. This process aligns with the requirements for preparing bauxite slurry in the Bayer process, eliminating excess water and maintaining liquid balance in the circulation system [1–3]. Generally, the modeling, optimization, and control of this process are based on the assumption that the process is in a steady or near-steady state. In recent studies, several advanced fault diagnosis techniques have leveraged both statistical methods and meta-heuristic optimization to address the complexities of industrial processes. A comprehensive analysis by Khan et al. provides a comparative overview of these techniques and highlights their respective strengths and limitations [4]. Additionally, adaptive metaheuristic optimization approaches, such as the one applied to the Tennessee Eastman process, have shown significant improvements in fault-tolerant control systems [5, 6]. However, fluctuations in fresh steam and raw liquid input, along with potential instrument faults or noise, can destabilize the evaporation process. This instability affects the relationship between input and output parameters, leading to inaccuracies in process modeling, parameter identification, optimization, and control. Therefore, steady-state detection (SSD) is crucial for data coordination [7–9], process modeling [10–12], performance evaluation [13], parameter identification, fault diagnosis [14–16], comprehensive optimization [17], and control [18] in the production process.

Our proposed method integrates advanced optimization techniques to enhance the scheduling of hybrid energy networks, building on the computational paradigm presented by Khalid et al. This approach effectively manages renewable energy uncertainties, improving overall system performance [19]. Additionally, the integration of event-triggered secure filtering techniques enhances the robustness and security of industrial systems against network attacks, aligning with the strategies developed by Basit et al. [20]. It is evident that during the process of steady-state detection, the mismatch in sampling signals is caused by the fact that the assumptions of the detection methods for various factors are not completely consistent during the detection process. In response to this issue, researchers both domestically and internationally have proposed steady-state detection methods from six major perspectives, including comprehensive multivariate detection and trend quantitative characterization. Commonly used steady-state detection methods include those based on mechanistic analysis, feature extraction, and statistical theory. Among these, combined statistical tests [21] (CST method), Mathematical Theory of Evidence [22] (MTE method), R test [23, 24], T test [25], and F test [26] are used to verify the results that need to be tested and to determine whether they have reached a steady state. Additionally, methods such as wavelet transform, neural networks, polynomial filtering, and fuzzy methods [27–34] are employed for feature extraction of measurement data, followed by establishing the representation trend through various test quantities. Meanwhile, the detection threshold is determined based on the collected data and engineering experience to ascertain whether the data to be tested is in a steady state. Steady-state detection methods based on statistical theory, trend extraction, and mechanistic analysis each have their advantages and disadvantages. For example, polynomial filtering and steady-state detection methods based on fuzzy sets can provide qualitative characterizations of whether a state is steady or not, but they do not offer quantitative indicators. Wavelet transform and polynomial filtering, both being signal processing-based filtering methods, have noise resistance capabilities.

The Combined Statistical Test (CST) and the Mathematical Theory of Evidence (MTE) based on statistical theory divide the data to be tested according to the average space and conduct a confidence level test on all the data to be tested. If the level of the tested data falls within the range of the confidence level, the condition is considered to be in a steady state; otherwise, it is considered to be in a non-steady state. Both the Combined Statistical Test (CST) and the Mathematical Theory of Evidence (MTE) are developed on the basis of random errors and are predicated on the assumption that they follow a normal distribution with an expectation of zero. Rhinehart and others believe that the R test method can be used without applying a time window to process data beforehand [35]. Instead, the ratio of variance estimates before and after filtering can be used directly to determine the steady state.

In addition to the above steady-state detection methods, the Bayer process for alumina production faces unique challenges. Actual production often involves manual sampling to determine the concentration of mother liquor at the evaporation outlet, and errors in this process can lead to anomalous data. Furthermore, on-site sensors and transmitters are subject to harsh conditions such as high temperatures, high pressures, and strong alkalinity, leading to sudden changes in conditions, equipment failures, and unreliable process data. This includes measurement errors, data loss, anomalies, and unmeasured variables, to which steady-state detection methods are particularly sensitive. Therefore, eliminating these anomalies is crucial for accurately reflecting the actual production process in steady-state detection. Additionally, the multi-effect falling film evaporation process involves numerous variables like temperature, pressure, flow, liquid level, and vacuum. Hence, single-variable steady-state detection cannot accurately represent the state changes in the evaporation process. Consequently, this paper introduces a method for adaptive steady-state detection based on multivariate data fusion in the evaporation process.

## 2 Evaporation process flow and analysis

### 2.1 Evaporation process flow

**Fig 1:**
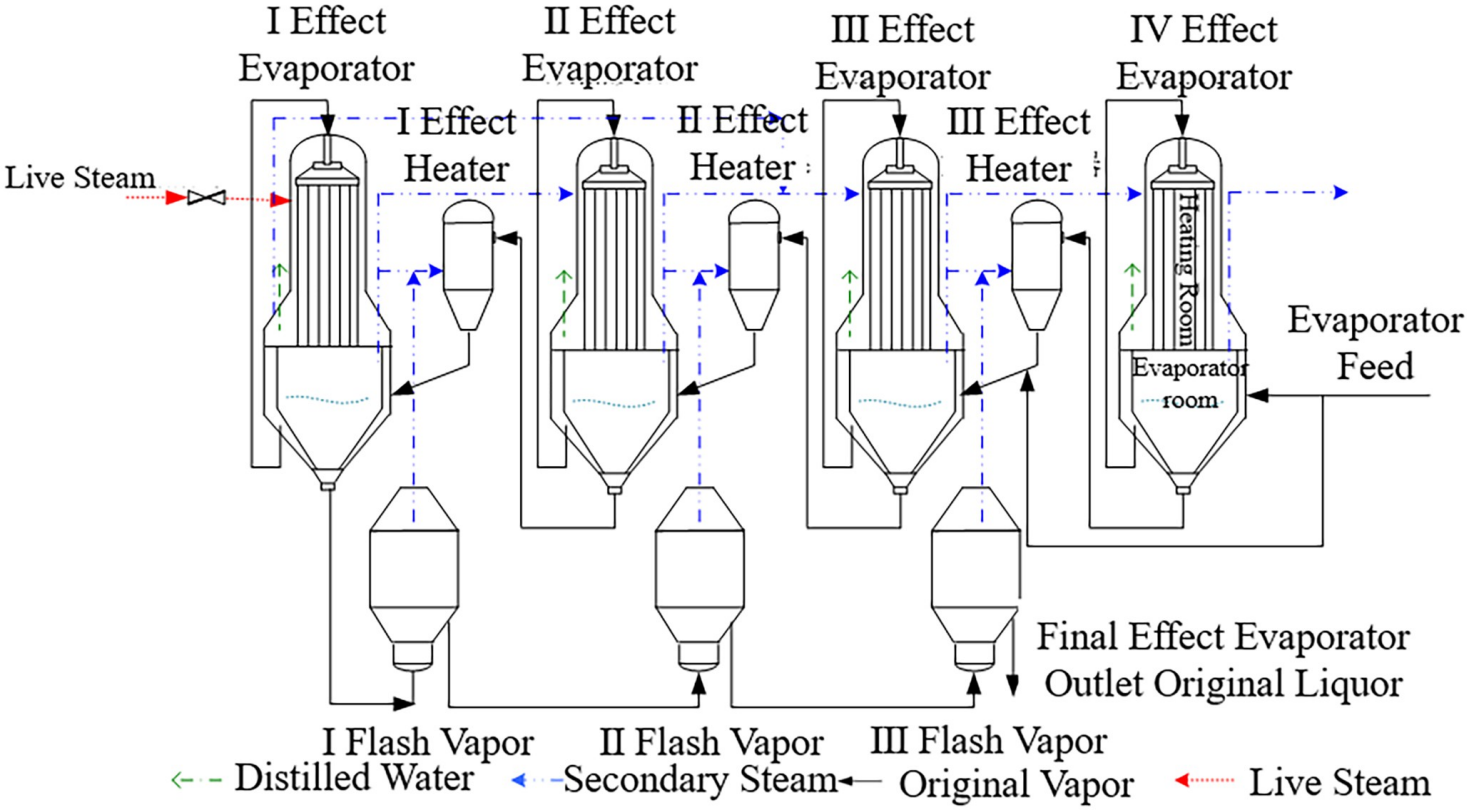
Process flow diagram of alumina production with quadruple-effect and triple flash evaporation.

The study focuses on the four-effect, three-flash falling film evaporation process in an alumina plant, as shown in Fig 1.

The four-effect, three-flash falling film evaporation process in alumina production primarily involves four evaporators, three preheaters, three flash evaporators, and condensate pumps. Based on the process flow of the four-effect countercurrent, three-stage flash distillation shown in Fig 1, the feed liquid is pumped to the fourth-effect evaporator. After heating in the respective evaporators, it undergoes three-stage flash vaporization for concentration, and then is output via a feed pump. In the first-effect evaporator, fresh steam from the thermal power plant heats the feed liquid. The heating source for the first to third-effect evaporators comes from the secondary steam generated by the previous effect. The secondary steam produced by the fourth evaporator is cooled and discharged through a condenser. The secondary steam from the first, second, and third-stage flash evaporators, along with the residual secondary steam from the first, second, and third-effect evaporators, heat the solutions in their respective preheaters. The condensate from the first four effects is discharged from the corresponding condensate evaporator, and the exhausted steam generated at the end of evaporation serves as a heat source for each effect. After cooling through condensate water, it is discharged via a water pump, ensuring smooth discharge of steam from the evaporation system [36].

### 2.2 Process analysis

The concentration of mother liquor at the outlet of the alumina evaporation process is a vital industrial metric. To meet the dissolution stage’s needs, the sodium hydroxide concentration in this mother liquor typically ranges between 160–170 g/L. Variations outside this range can affect the production efficiency of the dissolution process.

The concentration of the outlet mother liquor in the evaporation process is influenced by several factors. These include the parameters of the feed liquid entering the evaporation process, the parameters of the fresh steam, and the operational processes involved. Key factors impacting this concentration are the flow rate, temperature, and concentration of the feed liquid; the flow rate, temperature, and pressure of the fresh steam; scaling in the evaporators; and the vacuum level. These elements, based on the heat transfer mechanism in the evaporation process and practical production experiences, play significant roles in determining the concentration of the outlet mother liquor.

#### 2.2.1 Influence of the feed liquid parameters

**Fig 2:**
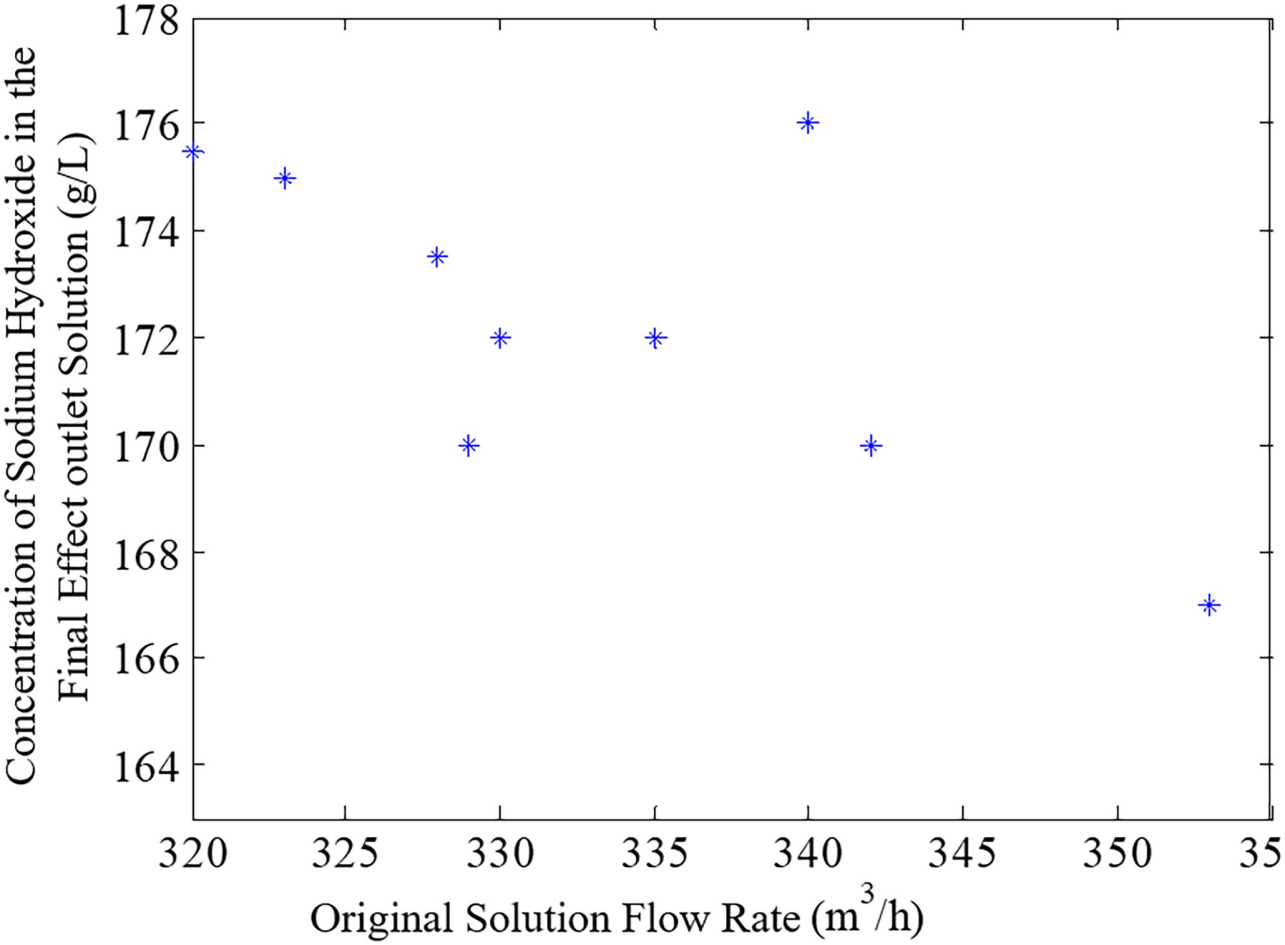
Relationship between the concentration of original liquor at the evaporation outlet and the flow rate of the original liquor.

During the entire evaporation process, assuming the fresh steam flow, temperature, and vacuum level of the evaporator remain constant, changes in the original liquid flow will directly affect the concentration of the mother liquor at the outlet. When the flow of the original liquid increases, the material flow rate accelerates, the film thickness grows, and the temperature rise within the time it takes for the material to flow from the top to the bottom of the tube decreases. Simultaneously, the liquid level in the evaporator rises, reducing the heat in the evaporator’s liquid and slowing the evaporation rate, resulting in a decreased concentration of the outlet mother liquor. Therefore, an excessive flow of the original liquid can cause the concentration of the outlet mother liquor to not reach the expected value. Conversely, when the flow of the original liquid decreases, the liquid level in the evaporator will drop, and the ample heat in the evaporator promotes the evaporation of the liquid, thereby increasing the concentration of the outlet mother liquor. However, a too low flow of the original liquid not only reduces the processing capacity of the process but also causes the material film to be too thin, forming dry scabs and reducing heat transfer efficiency. As shown in Fig 2, the impact of changes in the original liquid flow on the concentration of the evaporator outlet mother liquor is significant in the actual production process. The greater the original liquid flow, the lower the concentration of the outlet mother liquor, and vice versa; too low a flow affects the output. Therefore, in actual production, the flow of the original liquid must be controlled within a certain range.

**Fig 3:**
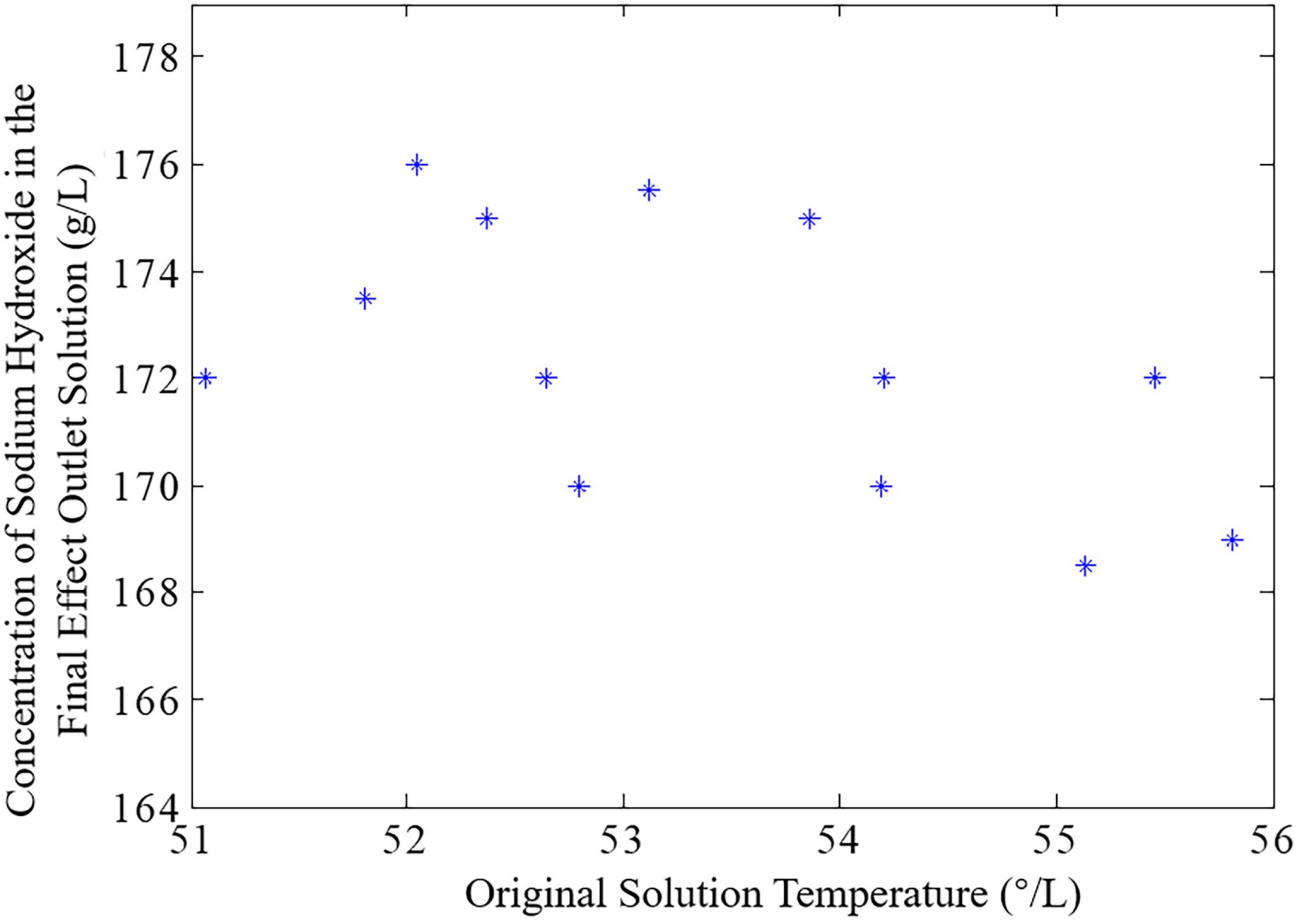
Relationship between the concentration of original liquor at the evaporation outlet and the temperature of the original solution.

Theoretically, when the flow rate of the feed liquid remains constant, a decrease in its temperature or concentration requires more heat to evaporate the water, thus reducing the concentration of the mother liquor at the evaporation outlet. Conversely, an increase in temperature or concentration leads to an opposite effect. However, in practical production, due to the strong non-linearity of the process and the intense coupling between multiple variables, the relationship between the feed liquid temperature and the concentration of the mother liquor at the evaporation outlet is neither strictly increasing nor decreasing, as illustrated in Fig 3.

#### 2.2.2 Impact of fresh steam parameters

An increase in the flow and temperature of fresh steam raises the heat transfer in the evaporator, leading to a higher concentration in the outlet mother liquor due to more heat absorption by the process fluid. However, an increase in steam flow also significantly raises energy costs, necessitating an optimal flow rate that meets concentration requirements without excessive steam use.

The pressure of fresh steam, when increased, also raises its temperature and flow, but this requires stronger equipment and considering cost factors, it’s generally advisable not to have excessively high steam pressure.

#### 2.2.3 Impact of evaporator scaling

The evaporation feed liquid is a high-viscosity solution, making it prone to crystallization of impurities. These crystals, if not promptly removed, deposit on the inner walls of heating tubes, forming scale over time.

The evaporative capacity of the evaporator is determined by the convective heat transfer rate equation, involving the heat transfer area, effective temperature difference, and heat transfer coefficient. The internal walls of the evaporator have minimal thermal resistance, but scaling significantly increases overall thermal resistance, affecting heat transfer. Scaling not only reduces the heat transfer coefficient but can also block the heating tubes. When scale thickness reaches 0.2mm, the heat transfer coefficient drops to about 57% of its value without scaling. At 0.5mm thickness, it decreases to around 40% or even lower. Therefore, increased scale thickness leads to greater thermal resistance, reducing evaporation efficiency and impacting the concentration of the outlet mother liquor.

**Fig 4:**
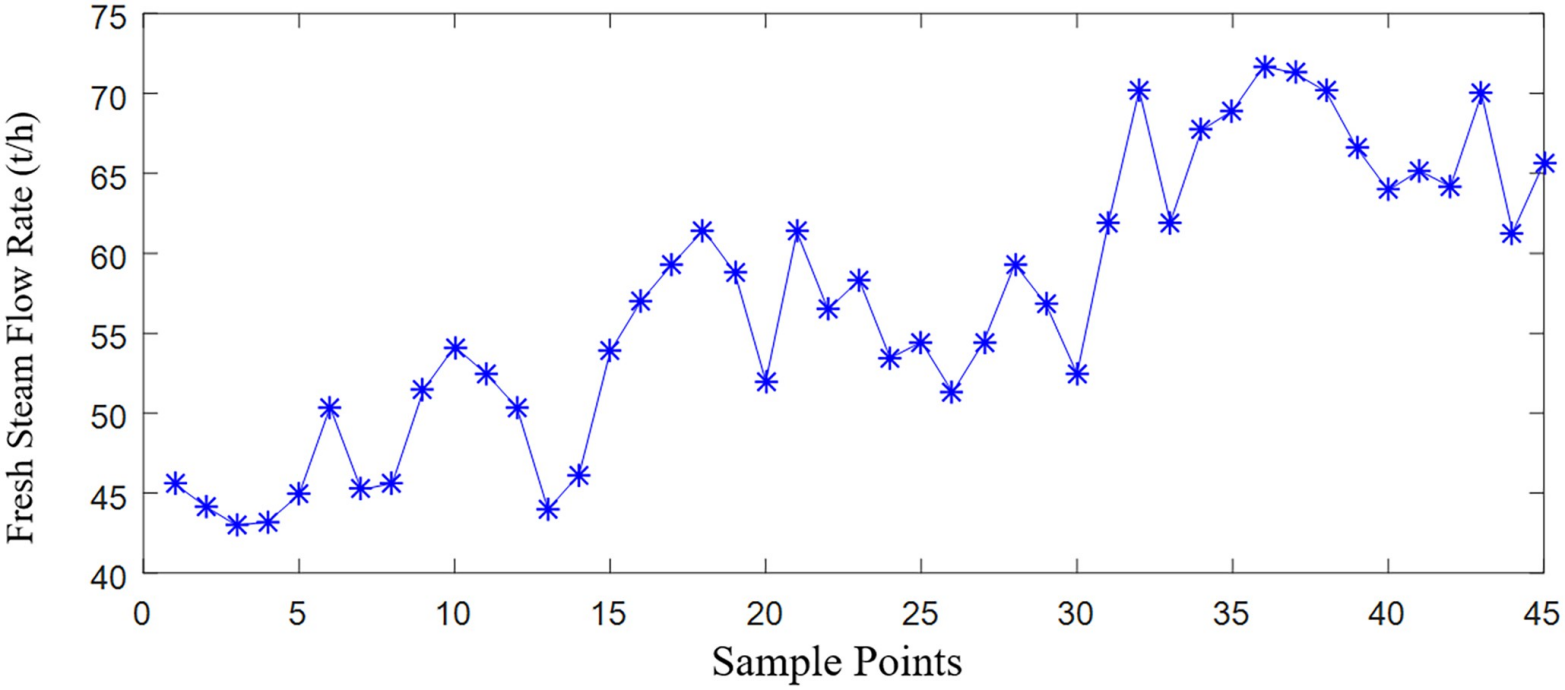
Changes in fresh steam flow during a pickling cycle.

In actual alumina evaporation production, equipment requires periodic shutdowns for acid washing to remove scale. The duration from one acid wash to the next is referred to as a washing cycle. During this cycle, as scaling and tube clogging become more severe, adjustments in the feed liquid flow and fresh steam usage are necessary to maintain the desired outlet mother liquor concentration, as shown in the provided data and illustrated in Fig 4.

In the later stages of an acid washing cycle, an evaporator with 700 tubes experiences nearly 50% tube blockage due to scaling. This highlights that scaling not only increases thermal resistance and reduces the heat transfer area, but also blocks the tubes, affecting the evaporator’s heat transfer capability. The higher the concentration of the evaporating mother liquor under the same temperature conditions, the poorer its flowability and the more likely it is to cause scaling. Therefore, keeping the concentration of the outlet mother liquor within a certain range slows down the scaling in the heating tubes and extends the operation cycle of the evaporation process.

The operating conditions in the actual alumina production evaporation process are complex and variable, with uncertainties such as variable parameters at the evaporator inlet, undetectable scaling in the evaporator, anomalous data values, and random noise on-site. These factors mean that when operating conditions change, the concentration of the outlet mother liquor may not achieve the desired stable target.

Given these challenges, the accuracy of steady-state detection in the alumina production evaporation process is crucial. It’s necessary to explore an adaptive steady-state detection strategy based on multivariate data fusion, which can handle the uncertainties of the evaporation process.

## 3 Adaptive steady-state detection in evaporation process based on multivariate data fusion

In the alumina evaporation process, numerous operational parameters are influenced by high temperature, strong alkali production environments, and other disturbances, potentially introducing errors in measurement data, as illustrated by Eq 1. To avoid incorrect and complex steady-state determinations, an adaptive steady-state detection method using data fusion of multivariate detection results is proposed. Its framework is outlined in Fig 3. Initially, variables impacting the outlet fluid concentration are preliminarily selected based on process analysis. Subsequently, variables with significant impact on outlet fluid concentration, such as fresh steam pressure, flow, and feed liquid flow, are chosen based on multivariate partial correlation analysis and practical production experience. The method then employs an adaptive Gaussian filter algorithm for error data filtering, followed by fidelity-preserving original signal processing. Steady-state detection is performed using a multivariate R-test method based on adaptive Gaussian filtering. Finally, the effectiveness of this method is validated using industrial field data. Our proposed method integrates advanced optimization techniques to enhance the scheduling of hybrid energy networks, building on the computational paradigm presented by Khalid et al. This approach effectively manages renewable energy uncertainties, improving overall system performance [19]. Additionally, the integration of event-triggered secure filtering techniques enhances the robustness and security of industrial systems against network attacks, aligning with the strategies developed by Basit et al [20].

### 3.1 Selection of steady-state detection variables in alumina plant

In the alumina factory’s four-effect countercurrent falling film evaporation process, the lengthy process flow and complex technological aspects lead to multiple factors affecting the outlet fluid concentration. Based on the previous analysis of the evaporation process, the initial selection of variables includes fresh steam flow (*X*_*l*_), fresh steam pressure (*X*_*p*_), fresh steam temperature (*X*_*T*_), feed liquid concentration (*S*_*c*_), feed liquid temperature (*S*_*t*_), feed liquid flow (*S*_*L*_), condensate water temperature (*CW*_*T*_), outlet fluid temperature (*L*_*T*_), vacuum degree (*VD*), secondary steam temperature (*SS*_*T*_), secondary steam pressure (*SS*_*P*_), and outlet fluid temperature (*L*_*C*_). We set Z as an array comprising these variables: [*X*_*l*_, *X*_*p*_, *X*_*T*_, *S*_*c*_, *S*_*T*_, *S*_*L*_, *CW*_*T*_, *L*_*T*_, *VD*, *SS*_*T*_, *SS*_*P*_, *L*_*C*_].

Next, a partial correlation analysis is used to filter out the variables with significant impacts on the outlet fluid concentration. The sample set of the influencing variables is defined as *U* = [*u*^1^, *u*^2^…, *u*^*m*^] ∈ *R*^*n*×*m*^, where n and m represent the number of samples and the dimension of variables, respectively. The original sample set is normalized to produce a specific result $\tilde{U}=[{\tilde{u}}^{1},{\tilde{u}}^{2}\ldots ,{\tilde{u}}^{m}]\in R^{n\times m}$. The partial correlation matrix is then defined as shown in Eq 1.

$$\begin{array}{c} M=[\begin{array}{cccc} q_{11} & q_{12} & \cdots & q_{1m}\\ q_{21} & q_{22} & \cdots & q_{2m}\\ \vdots & \vdots & \cdots & \vdots \\ q_{m1} & q_{m2} & \cdots & q_{mm} \end{array}] \end{array}$$ (1)

Here: $q_{ij}=\theta_{ij}/\sqrt{\theta_{ii}\theta_{ij}},i=1,2,\ldots ,m,j=1,2,\ldots ,m,\theta_{ij}=\sum_{k=1}^{n}{\tilde{u}}_{k}^{i}{\tilde{u}}_{k}^{j}-\frac{1}{n}\sum_{k=1}^{n}{\tilde{u}}_{k}^{i}\sum_{k=1}^{n}{\tilde{u}}_{k}^{j}$.

The partial correlation coefficient between related variable ${\tilde{u}}^{i}$ and related variable ${\tilde{u}}^{j}$ is as given in Eq 2.

$$\begin{array}{c} {\tilde{q}}_{ij}=-\delta_{ij}/\sqrt{\delta_{ii}\delta_{ij}},i=1,2,\ldots ,m,j=1,2,\ldots ,m \end{array}$$ (2)

Here: Element *δ*_*ij*_ refers to an element within the invertible matrix ***M***^−1^ of the partial correlation matrix ***M***.

$$\begin{array}{c} M^{-1}=[\begin{array}{cccc} \delta_{11} & \delta_{12} & \cdots & \delta_{1m}\\ \delta_{21} & \delta_{22} & \cdots & \delta_{2m}\\ \vdots & \vdots & \cdots & \vdots \\ \delta_{m1} & \delta_{m2} & \cdots & \delta_{mm} \end{array}] \end{array}$$ (3)

100 sets of industrial field data samples were selected to calculate the partial correlation coefficient matrix among 12 variables, as shown in Table 1.

**Table 1:** Partial correlation coefficient matrix of influencing variables.

	*X*_*l*_	*X*_*p*_	*X*_*T*_	*S*_*c*_	*S*_*T*_	*S*_*L*_	*CW*_*T*_	*L*_*T*_	VD	*SS*_*T*_	*SS*_*P*_	*L*_*C*_
*X*_*l*_	1.000											
*X*_*p*_	-0.680	1.000										
*X*_*T*_	0.127	0.010	1.000									
*S*_*c*_	0.412	0.302	-0.319	1.000								
*S*_*T*_	-0.518	-0.549	0.442	0.774	1.000							
*S*_*L*_	0.563	0.635	-0.226	-0.553	0.857	1.000						
*CW*_*T*_	0.261	0.064	-0.119	-0.381	0.244	0.082	1.000					
*L*_*T*_	0.004	0.094	0.053	-0.080	-0.071	0.023	0.023	1.000				
VD	-0.508	-0.373	0.175	0.528	-0.606	0.439	0.842	-0.087	1.000			
*SS*_*T*_	-0.052	0.237	0.238	0.405	0.000	-0.336	0.617	0.111	-0.277	1.000		
*SS*_*P*_	0.382	0.291	-0.029	-0.367	0.407	-0.246	-0.842	0.061	0.919	0.323	1.000	
*L*_*C*_	0.360	0.341	0.058	-0.067	0.186	-0.331	-0.004	-0.010	0.147	0.115	-0.078	1.000

Based on the data analysis results from Table 1 and actual production conditions, three variables with significant impact have been selected for steady-state detection. These are the fresh steam flow (with a partial correlation coefficient of 0.360), fresh steam pressure (partial correlation coefficient of 0.341), and feed flow rate (partial correlation coefficient of 0.331).

### 3.2 The adaptive Gaussian filtering algorithm

The Gaussian filtering algorithm involves convolving the original signal with a Gaussian kernel function, also known as a Gaussian filter. The one-dimensional Gaussian function is set as:

$$\begin{array}{c} g\left(t,\sigma \right)=\frac{1}{\sqrt{2\pi }\sigma }\text{exp}(-\frac{t^{2}}{2\sigma^{2}}) \end{array}$$ (4)

Its first derivative is:

$$\begin{array}{c} g^{\prime }\left(t,\sigma \right)=\frac{-t}{\sqrt{2\pi }\sigma^{3}}\text{exp}(-\frac{t^{2}}{2\sigma^{2}}) \end{array}$$ (5)

In the formula, *g*′(*t*, *σ*) represents the Gaussian filter, and *σ* is the standard deviation of the Gaussian kernel function. The function’s output after applying the Gaussian filter *f*(*t*) is expressed as:

$$\begin{array}{c} S\left(t,\sigma \right)=f\left(t\right)*g^{\prime }\left(t,\sigma \right) \end{array}$$ (6)

Let m represent the sampling time, *f*(*m*) the historical signal of measurement data, *f*_*k*+1_(*m*) the output value after the (k+1)th iteration, *w*_*k*_(*m* + *j*, *σ*) the weights of each point within the time window, and (2*M* + 1) the length of the filtering window. The relationship is expressed as:

$$\begin{array}{c} f_{k+1}\left(m\right)=\frac{\sum_{j=-M}^{M}f_{k}\left(m+j\right)w_{k}\left(m+j,\sigma \right)}{\sum_{j=-M}^{M}w_{k}\left(m+j,\sigma \right)} \end{array}$$ (7)

The filtering weights are adaptively adjusted based on the derivative at the center point of the template, as follows:

$$\begin{array}{c} w_{k}\left(m,\sigma \right)=\text{exp}[-\frac{{\left|f_{k}^{\prime }\left(m\right)\right|}^{2}}{2\sigma^{2}}] \end{array}$$ (8)

In this formula, *σ* represents a constant parameter, k the iteration number, and $f_{k}^{\prime }\left(m\right)$ the first-order derivative of the signal value *f*_*k*_(*m*), which can be expressed as:

$$\begin{array}{c} f_{k}^{\prime }\left(m\right)=\frac{1}{2}[f_{k}\left(m+1\right)-f_{k}\left(m-1\right)] \end{array}$$ (9)

The ratio of $f_{k}^{\prime }\left(m\right)$ to the standard deviation of the Gaussian function determines the weight *w*_*k*_(*m*, *σ*). After smoothing the noise in the signal, the preservation level of the abrupt point information can be controlled by changing the value of *σ*. Optimal filtering is achieved when the parameter takes the value of $\sigma \approx f_{k}^{\prime }\left(m\right)$.

**Fig 5:**
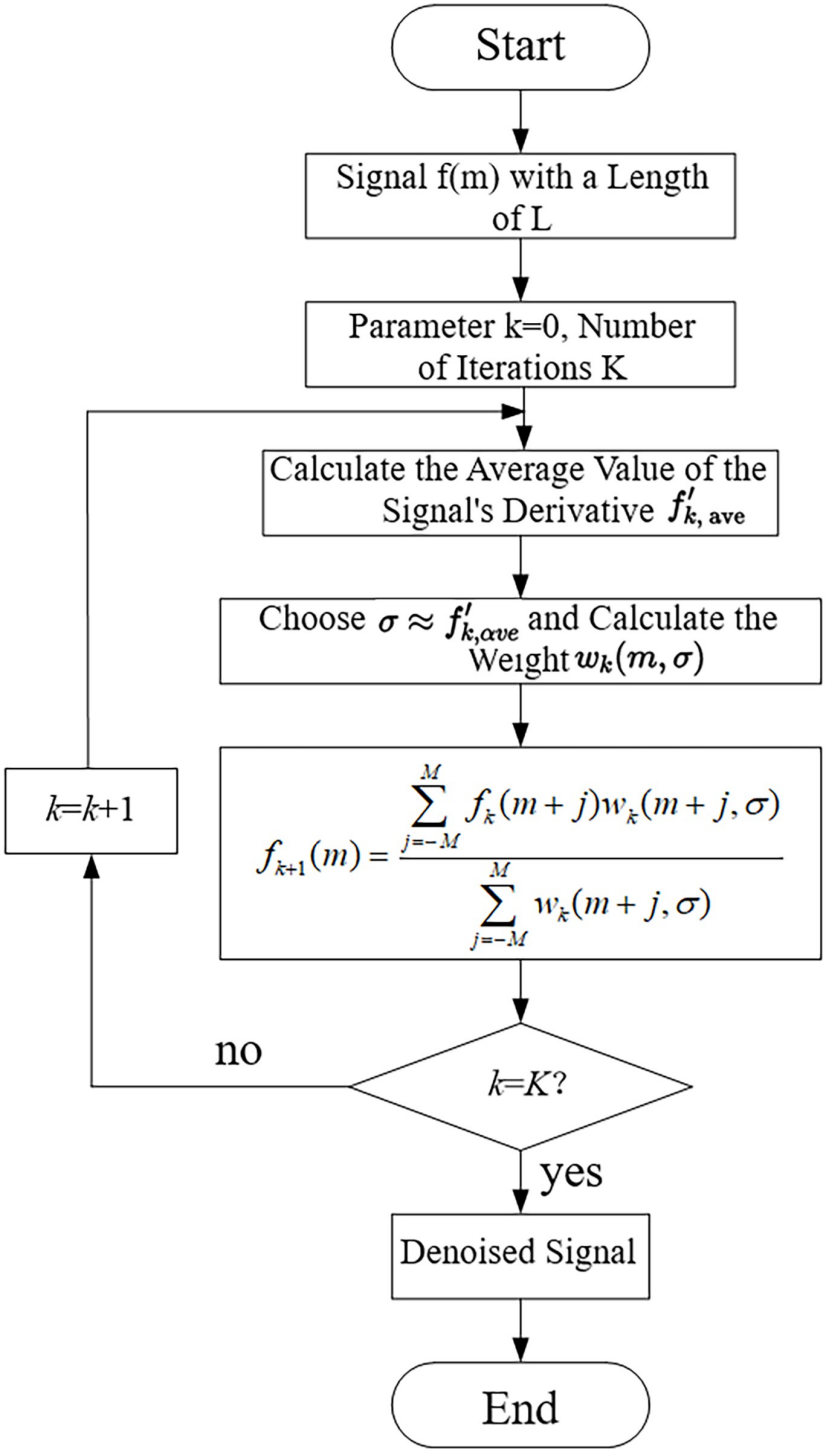
Specific process of the adaptive denoising algorithm based on Gaussian filter.

The process of the adaptive noise reduction algorithm based on the Gaussian filter is illustrated in Fig 5. Filtering is performed using a 1 × 5 template, setting the parameter M to 2 according to the size of the filtering window. The specific steps of the adaptive noise reduction algorithm are as follows:

1. Determine the number of signal sampling points *f*(*m*).
2. Define the number of iterations as K, initially assuming *k* = 0.
3. Calculate the first derivative of the signal $f_{k}^{\prime }\left(m\right)$, and calculate the average of all derivatives $f_{k,\;\text{ave}\;}^{\prime }$.
4. Set a certain parameter $\sigma \approx f_{k,ave}^{\prime }$ and calculate the weights *w*_*k*_(*m*, *σ*) for each signal point for denoising, as per Eq (5).

The iteration stops when the iteration count k equals K. If not, update k into *k* = *k* + 1, replace the original data with the newly obtained data, and repeat step 3.

### 3.3 R-test method

The measurement model for the variable x is defined as follows:

$$\begin{array}{c} \tilde{x}\left(t\right)=x\left(t\right)+e\left(t\right)+\gamma \left(t\right) \end{array}$$ (10)

In this equation, $\tilde{x}\left(t\right)$ represents the measured value; *x*(*t*) is the true value; *e*(*t*) indicates the measurement noise; and *γ*(*t*) signifies accidental error.

The R-test method, also known as the variance test method, uses variance to represent the degree of deviation. The variable’s variance is calculated to construct a steady-state detection statistic for making steady-state judgments. Suppose the measurement value of the time series X at the i-th moment is *x*_*i*_. The first-order filtered value at the i-th moment *x*_*f*,*i*_ is represented as shown in Eq 11, where the subscript f denotes that the variable x has undergone filtering. The mean square deviation of the measurement value at moment i *x*_*i*_ and the first-order filtered value at moment i-1 *x*_*f*,*i*−1_ is calculated using Eq 12, denoted as $u_{f,i}^{2}$. The squared deviation of the measurement value at a given moment *x*_*i*_ and the previous sampling moment’s measurement value *x*_*i*−1_ is calculated using Eq 13, denoted as $\delta_{f,i}^{2}$, with the filter coefficient being *λ*_*n*_ ∈ (0, 1](*n* = 1, 2, 3).

$$\begin{array}{c} x_{f,i}=\lambda_{1}x_{i}+(1-\lambda_{1})x_{f,i-1} \end{array}$$ (11)

$$\begin{array}{c} v_{f,i}^{2}=\lambda_{2}{\left(x_{i}-x_{f,i-1}\right)}^{2}+(1-\lambda_{2})u_{f,i-1}^{2} \end{array}$$ (12)

$$\begin{array}{c} \delta_{f,i}^{2}=\lambda_{3}{\left(x_{i}-x_{i-1}\right)}^{2}+(1-\lambda_{3})\delta_{f_{i,i-1}}^{2} \end{array}$$ (13)

Using $u_{f,i}^{2}$ and $\delta_{f,i}^{2}$, the estimated values of variance can be calculated as shown in Eq 14.

$$\begin{array}{c} s_{1,i}^{2}=\frac{2-\lambda_{1}}{2}u_{f,i}^{2},\;s_{2,i}^{2}=\frac{1}{2}\delta_{f,i}^{2} \end{array}$$ (14)

The ratio of $s_{1,i}^{2}$ to $s_{2,i}^{2}$ is denoted as the statistical quantity R, as shown in Eq 15.

$$\begin{array}{c} R_{i}=\frac{s_{1,i}^{2}}{s_{2,i}^{2}}=\frac{(2-\lambda_{1})v_{f,i}^{2}}{\delta_{f,i}^{2}} \end{array}$$ (15)

Through theoretical analysis and experimental simulation, Rhinehart and others [24] determined that when the confidence level is *α*_*i*_, the optimal threshold *R*_crit_ and the filtering coefficient *λ*_*i*_ are established. By comparing the values of *R*_*i*_ and *R*_crit_, the steady-state condition of the process variable can be judged. When *R*_*i*_ > *R*_*crit*_, it represents that the process is in a steady state, denoted as *SS*_*i*_ = 1. If *R*_*i*_ ≤ *R*_crit_, it indicates that the process variable is in a non-steady state, denoted as *SS*_*i*_ = 0.

The R test method is used for steady-state detection of individual variables, whereas for assessing the stability of a system, steady-state detection of multiple variables is required. If any one of the variables is in an unstable state during testing, the entire system is considered to be in a non-steady state; conversely, it is deemed stable. Based on this concept, a marker as shown in Eq 16 is constructed to characterize the process state:

$$\begin{array}{c} SS_{\text{process}\;}=\prod_{i=1}^{N}SS_{i} \end{array}$$ (16)

If the process is in a stable state and the changes in the process variables are independent of each other, then:

$$\begin{array}{c} P(SS_{\text{process}\;}=1)=\prod_{i=1}^{N}P(SS_{i}=1)=\prod_{i=1}^{N}(1-\alpha_{i}) \end{array}$$ (17)

$$\begin{array}{c} 1-\alpha_{\text{process}\;}=\prod_{i=1}^{N}(1-\alpha_{i}) \end{array}$$ (18)

From this, the individual confidence level for each variable can be determined based on the steady-state confidence of the process, as shown in:

$$\begin{array}{c} \alpha_{i}=1-\sqrt[{N}]{1-\alpha_{\text{process}\;}} \end{array}$$ (19)

Here, P represents probability, *α*_process_ is the overall confidence of the multivariable process, and N is the number of process variables. Steady-state detection of a process integrating multiple variables can be achieved according to Eq 16.

## 4 Application of simulation and analysis

New steam serves as the heat source for the evaporation production process. When the flow rate of new steam is too high, it ensures the first-effect evaporation capacity, but a portion of the steam will be carried away with the condensed water, leading to wastage. Conversely, if the flow rate is too low, the liquid feed cannot be sufficiently concentrated.

Since the temperature of the new steam cannot be directly controlled, the evaporation process generally adjusts its temperature indirectly by controlling the pressure of the new steam. When the steam pressure increases, the temperature also increases, significantly improving the heating capacity, but often resulting in superheated steam. Steam may condense into water droplets along the outer wall of the heating pipe, increasing heat transfer resistance. Furthermore, for the entire evaporation system, from the first-effect to the sixth-effect, it is a process of gradually decreasing pressure. If the pressure of the new steam is unstable, it can lead to changes in the system’s operating conditions. Therefore, the control of new steam pressure is particularly crucial.

The flow of the original solution runs through the entire evaporation process. As analyzed earlier, when the flow rate of new steam remains constant, the concentration of the outlet mother liquor varies with changes in the feed flow rate.

In the Bayer process for aluminum oxide evaporation, the variables for process steady-state detection are selected as new steam flow rate, new steam pressure, and original solution flow rate. Test data consists of 1080 sets of sampling data collected during continuous operation of the aluminum oxide production evaporation process over 36 hours. The test data was collected from an aluminum oxide plant during continuous operation over 36 hours, from March 5, 2022, to March 7, 2022, resulting in 1080 sets of sampling data. The steady-state detection method used is a multivariate fusion process based on the R test method with a confidence level set at 0.05. Reference provides a recommended critical value table, selecting a filter coefficient of *λ*_1_ = 0.2, *λ*_2_ = 0.1, *λ*_3_ = 0.01 and detection threshold of *R*_*crit*_ = 1.44. For the 1080 sample data points of the three variables, both median filtering and R test method based on adaptive Gaussian filtering are applied for steady-state detection, and both methods use a template of 1 × 5.

**Fig 6:**
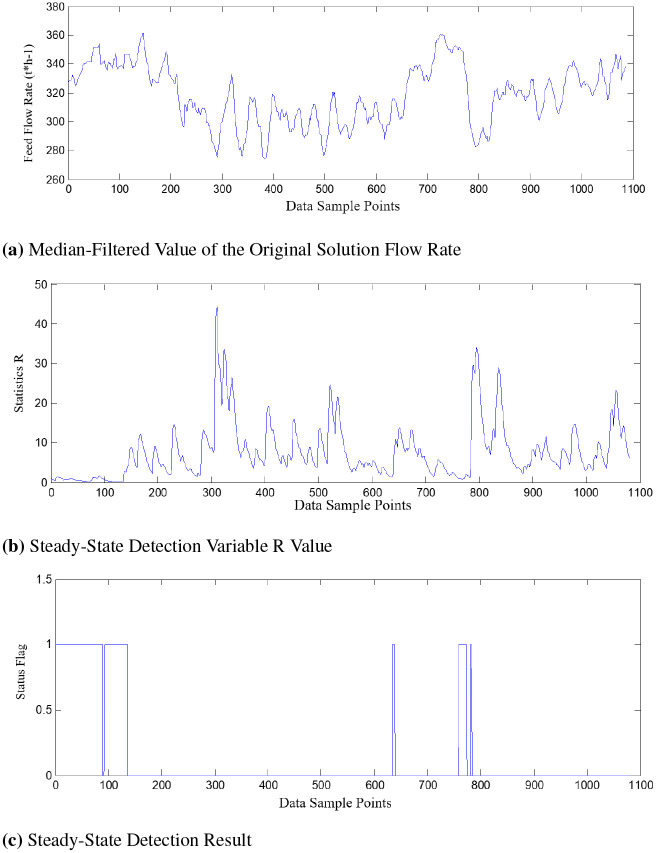
Steady-state detection based on median filtering. (a) Median-Filtered Value of the Original Solution Flow Rate. (b) Steady-State Detection Variable R Value. (c) Steady-State Detection Result.

**Fig 7:**
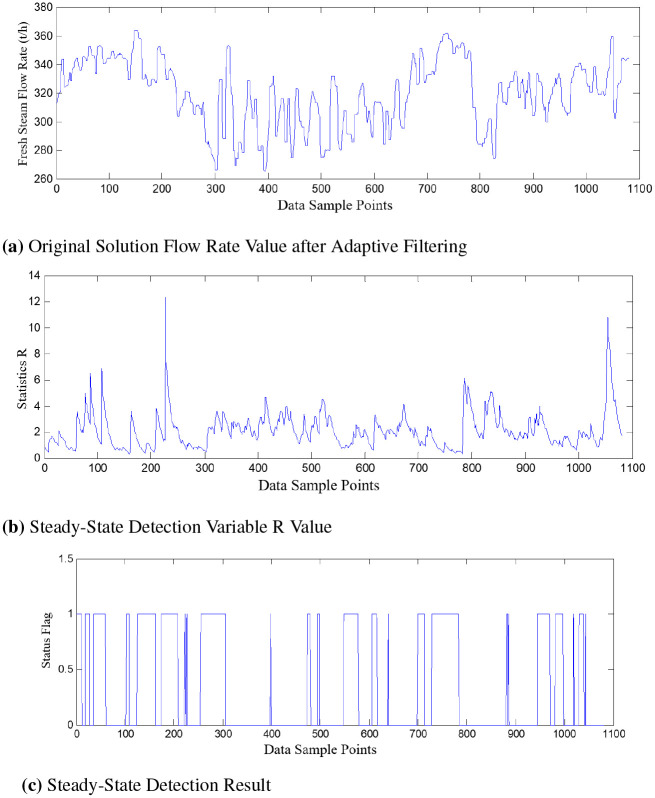
Steady-state detection based on adaptive filtering. (a) Original Solution Flow Rate Value after Adaptive Filtering. (b) Steady-State Detection Variable R Value. (c) Steady-State Detection Result.

Figs 6 and 7 represent the test results of steady-state detection using the two methods, where: Fig 6a represents the data processed using different filtering methods. Fig 6b shows the change in the trend of the steady-state detection variable R values at corresponding time points. Fig 6c represents the judgment results after steady-state detection based on the threshold. The region where the state variable is 1 corresponds to the steady-state region, while the region where the state variable is 0 represents the non-steady-state region.

Comparing the data curves of the filtered original solution flow rate in Figs 6a and 7a, it can be observed that the Gaussian filter method is superior to the median filter method.

**Fig 8:**
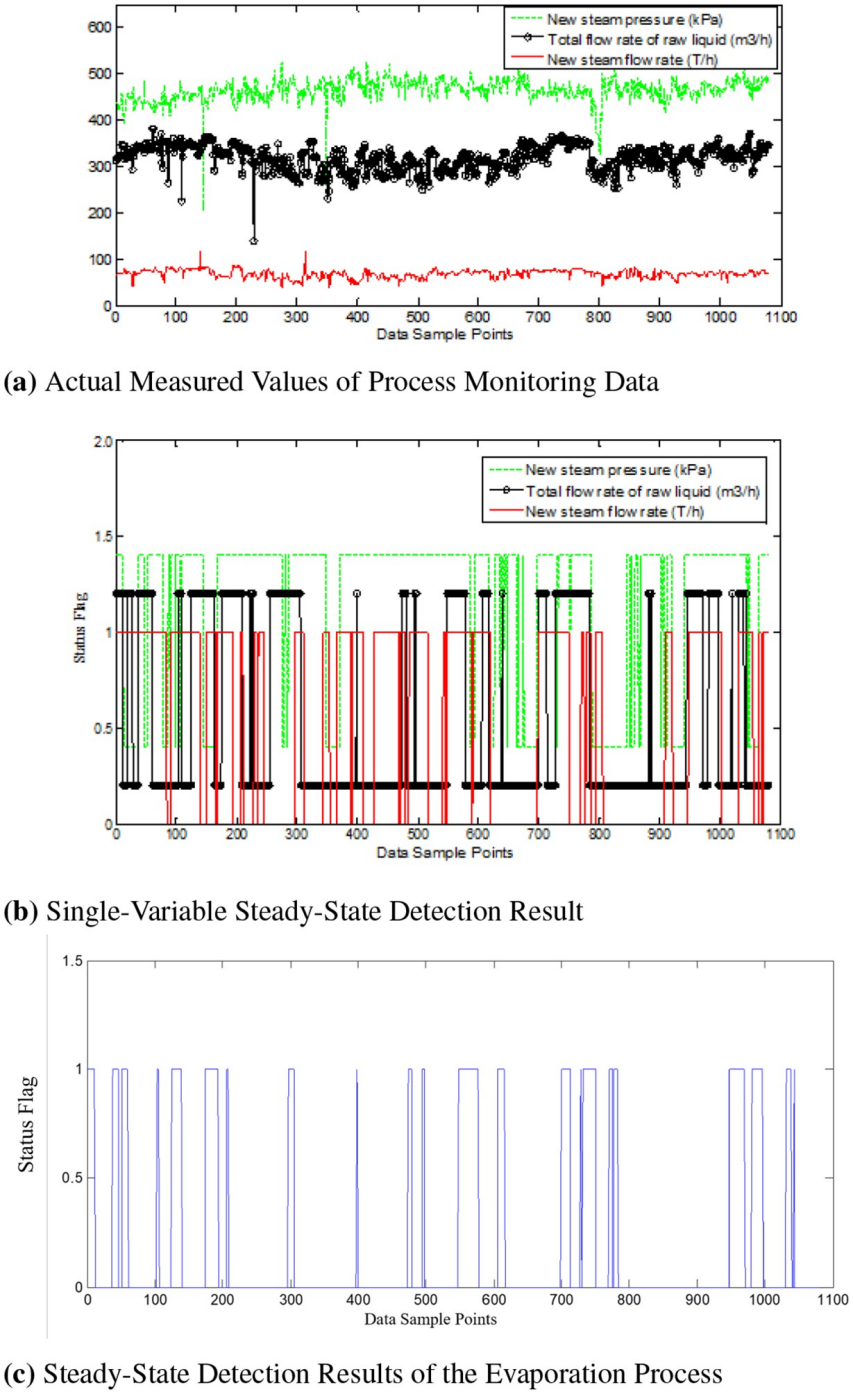
Evaporation process steady-state detection. (a) Actual Measured Values of Process Monitoring Data. (b) Single-Variable Steady-State Detection Result. (c) Steady-State Detection Results of the Evaporation Process.

In Fig 8a and 8b, dashed lines represent the new steam pressure (in kPa) and steady-state detection results. Solid lines without circles represent the new steam flow rate (in T/h) and steady-state detection results, while solid lines with circles represent the total flow rate of the original solution (in m^3^/h) and steady-state detection results. In Fig 8b and 8c, non-zero vertical coordinates indicate that the system is in a steady-state at that moment (to differentiate between the three detection results, two of the single steady-state detection results are shifted upward by 0.2 and 0.4, where 0 represents a non-steady-state condition at the corresponding time). Fig 8 demonstrates the ability to make correct judgments about the actual operating conditions when various process variables exhibit dynamic changes.

## 5 Conclusion

The online monitoring system for the aluminum oxide evaporation process stores a vast amount of historical data. These measurement data accurately reflect the production operation status. Extracting steady-state data from historical data can provide a wealth of information for modeling soft measurements in the evaporation process. Due to the inability to directly measure parameters of intermediate equipment in the evaporation process and the presence of noise and gross errors in the measured parameters, the production process becomes unstable. This paper proposes an adaptive steady-state detection method for the evaporation process based on the fusion of multivariate data. Initially, a Gaussian filter-based adaptive denoising algorithm is applied to eliminate faulty data from selected variables. After preserving the fidelity of the original signals, steady-state detection is performed using the multivariate R test method based on adaptive Gaussian filtering. Finally, the effectiveness of the proposed steady-state detection method is validated using actual production data. This method lays the foundation for modeling, optimization, and control of the aluminum oxide production evaporation process. The implementation of our method in real-world scenarios demonstrates significant improvements in the reliability and efficiency of hybrid energy network scheduling. These findings align with the results obtained by Khalid et al., highlighting the advantages of addressing renewable energy uncertainties in scheduling strategies. Furthermore, the enhancements in system robustness and security through event-based filtering, as discussed by Basit et al., underscore the effectiveness of our approach in managing network attacks and switching topologies.

While our approach has demonstrated significant improvements, it is important to acknowledge several potential limitations and assumptions. First, the accuracy of steady-state detection relies heavily on the quality of input data. The presence of noise and gross errors in the data can still pose challenges despite the use of advanced filtering techniques [37, 38]. Second, the proposed method assumes that the underlying statistical properties of the process variables remain consistent over time. Any significant changes in the process dynamics may affect the performance of the detection algorithm. Moreover, our method primarily focuses on steady-state detection and may not be directly applicable to processes with highly dynamic or transient behaviors. The adaptive nature of the Gaussian filtering algorithm helps to mitigate some of these issues, but further improvements are needed to enhance its robustness.

Future research can explore several avenues to address these limitations and enhance the proposed method. One potential direction is to develop more advanced denoising algorithms that can better handle non-stationary noise and abrupt changes in process variables. Additionally, integrating machine learning techniques, such as anomaly detection and predictive modeling, can further improve the accuracy and robustness of steady-state detection. Another area of research is the application of the proposed method to other industrial processes with different characteristics and challenges. This can help to generalize the approach and validate its effectiveness across a wider range of applications. Furthermore, developing real-time implementation strategies and optimizing computational efficiency will be crucial for deploying the method in industrial settings.

Finally, collaboration with industry partners can provide valuable insights and feedback, facilitating the continuous improvement and practical adoption of the method. By addressing these potential limitations and exploring new research directions, we can further enhance the reliability and applicability of steady-state detection techniques in industrial processes.

## Supporting information

S1 Data (PDF)
