# Supplementary material for: Steady-state detection of evaporation process based on multivariate data fusion
Source: PLoS One. 2024 Sep 6;19(9):e0309652. doi: 10.1371/journal.pone.0309652 (PMC11379215; doi:10.1371/journal.pone.0309652)
Supplement: S1 Data — (PDF) [file pone.0309652.s001.pdf]

New steam pressure（kPa）	Total flow rate of raw liquid（m^3/h）	New steam flow rate（T/h）
437.7367779	312.5023522	62.76168132
435.2449177	317.1456919	64.61179891
434.4142976	316.2505903	67.31581693
426.938717	315.8030395	67.88508388
422.7856166	320.4463791	66.4619165
433.5836775	322.4044139	68.16971735
421.1243764	319.3275021	69.59288473
427.769337	320.3904352	69.59288473
456.8410395	323.858954	67.88508388
448.5348388	346.9078207	67.74276714
454.3491793	344.3344035	70.73141863
423.6162367	343.4393019	68.7389843
405.3425951	343.3833581	69.45056799
406.1732152	344.2225158	71.44300232
389.5608137	316.0827587	80.40895679
423.6162367	324.4743364	73.86238686
437.7367779	318.8799513	68.45435083
449.3654589	317.7610742	69.45056799
459.3328998	323.858954	72.01226927
456.0104195	325.6491573	71.15836884
459.3328998	324.5302802	71.7276358
455.1797994	336.3344328	71.15836884
446.0429786	336.0547136	70.58910189
454.3491793	335.5512189	71.01605211
446.8735987	324.4183925	69.16593452
446.8735987	321.2855369	66.60423324
446.0429786	332.865914	66.31959976
460.9941399	293.3136113	41.12953719
469.3003406	339.8029516	45.25672259
446.0429786	334.1526226	62.19241437
418.6325162	320.7820422	72.58153622
441.8898782	335.7190505	65.89264955
446.0429786	336.1106574	66.17728303
434.4142976	338.90785	69.02361778
433.5836775	344.5022351	69.30825125
445.2123585	343.3274142	69.45056799
446.0429786	344.2784597	69.59288473
450.196079	344.2784597	71.30068558
446.8735987	343.9427966	70.44678516
450.196079	344.6141228	71.15836884
446.0429786	338.8519061	69.59288473
438.567398	344.0546843	73.86238686
442.7204983	338.4602992	75.71250445
437.7367779	335.7190505	75.57018771
436.0755377	335.7190505	75.1432375

422.7856166	335.6631066	73.00848643
481.7596417	335.6631066	79.55505637
437.7367779	329.7330584	75.28555424
437.7367779	346.1805506	76.70872161
438.567398	348.5861362	75.1432375
436.9061578	349.5371817	73.29311991
437.7367779	350.2644517	73.00848643
440.2286381	343.1595826	74.14702034
437.7367779	344.8378982	74.0047036
435.2449177	344.7819543	73.43543665
432.7530575	344.7819543	73.86238686
436.0755377	335.8309382	73.57775339
434.4142976	336.8938713	73.90508188
421.1243764	340.9777725	76.99335509
422.7856166	335.7190505	72.72385296
427.769337	380.8097944	74.0047036
425.2774768	380.8097944	74.28933707
416.9712761	380.8097944	70.87373537
443.5511184	357.7049839	76.13945466
432.7530575	347.0756522	81.40517396
415.3100359	347.0756522	78.13188899
422.7856166	345.6770559	75.42787098
433.5836775	346.5721576	74.43165381
436.9061578	347.0756522	75.42787098
434.4142976	345.9008314	73.43543665
438.567398	341.4253233	73.15080317
433.5836775	331.1316547	70.16215168
437.7367779	335.7190505	71.30068558
436.0755377	352.5022058	71.86995253
431.0918173	370.1245189	74.0047036
451.026699	306.4604163	59.48839635
462.65538	295.9429723	62.19241437
438.567398	329.117676	65.46569934
408.6650754	352.4462619	60.19998004
475.1146811	353.1175881	48.81464103
433.5836775	352.6140935	59.20376288
432.7530575	351.663048	68.45435083
450.196079	352.5581496	78.70115594
431.9224374	352.6140935	82.11675765
439.398018	351.7749357	82.54370786
434.4142976	352.8378689	81.97444091
433.5836775	263.4395948	81.83212417
428.5999571	335.7190505	81.12054048
435.2449177	338.8519061	80.55127353
407.8344553	340.026727	75.71250445
445.2123585	341.2574917	77.70493878

433.5836775	340.9218286	78.41652247
445.2123585	340.4742778	82.97065807
453.5185593	344.2225158	79.98200658
454.3491793	345.0057297	80.83590701
455.1797994	359.942738	78.41652247
443.5511184	330.236553	73.86238686
459.3328998	343.6630773	75.57018771
423.6162367	341.6490987	70.01983494
452.6879392	343.8868527	72.58153622
455.1797994	344.4462912	73.29311991
446.8735987	344.4462912	73.15080317
453.5185593	343.4393019	74.28933707
445.2123585	346.6281014	75.99713793
453.5185593	353.4532512	79.27042289
467.6391005	351.5511603	78.98578942
483.4208818	359.8867941	81.83212417
470.1309607	360.8378396	79.98200658
470.9615808	225.3418322	78.98578942
460.9941399	321.0058176	77.70493878
459.3328998	339.4113446	77.13567183
442.7204983	344.4462912	59.91534657
441.8898782	343.4393019	76.85103835
447.7042188	345.285449	77.4203053
460.9941399	347.1875399	75.99713793
459.3328998	346.6840453	75.85482119
456.8410395	348.9217993	78.13188899
462.65538	346.8518768	78.98578942
461.82476	346.8518768	78.41652247
456.8410395	346.8518768	79.69737311
460.1635198	346.404326	80.55127353
452.6879392	350.152564	74.71628729
439.398018	351.8868234	81.26285722
456.8410395	351.4952165	73.72007012
452.6879392	343.8868527	76.13945466
441.8898782	343.9427966	79.69737311
457.6716596	343.9427966	79.83968984
453.5185593	344.0546843	80.26664006
459.3328998	345.6211121	79.69737311
451.8573191	343.2714703	80.12432332
455.1797994	342.5442003	80.97822375
456.8410395	345.3973367	79.27042289
452.6879392	344.0546843	80.12432332
452.6879392	344.0546843	78.13188899
450.196079	324.4743364	78.84347268
456.0104195	324.4743364	80.69359027
456.8410395	323.858954	76.99335509

474.2840611	327.7750236	76.42408814
468.4697205	330.4603284	75.71250445
460.9941399	330.4603284	117.1266751
465.1472403	349.8728448	77.98957225
445.2123585	351.8868234	78.98578942
445.2123585	349.0896309	78.84347268
475.1146811	343.1036388	83.25529155
207.655018	344.0546843	82.11675765
436.0755377	355.2434545	77.27798857
447.7042188	364.1944707	78.84347268
446.0429786	363.6350322	79.55505637
437.7367779	364.0266391	83.25529155
441.8898782	363.5790883	83.68224176
441.0592582	363.1315375	82.6860246
422.7856166	363.690976	81.12054048
441.0592582	363.9147514	82.25907438
451.026699	363.690976	81.5474907
456.0104195	358.0965909	80.12432332
465.9778603	358.4881978	72.01226927
466.8084804	357.8168716	64.04253196
471.7922008	357.4252647	68.31203409
458.5022797	357.8728155	68.45435083
459.3328998	358.040647	73.29311991
440.2286381	356.8098823	75.71250445
440.2286381	324.8659433	77.84725552
464.3166202	290.9639696	66.17728303
472.6228209	290.4045311	64.61179891
480.9290216	324.8659433	61.62314742
480.9290216	334.4323419	56.21511139
476.7759213	335.0477242	56.07279465
464.3166202	341.2574917	62.33473111
454.3491793	341.8169302	63.18863153
455.1797994	326.7120904	63.75789848
455.1797994	330.5722161	64.61179891
456.0104195	330.0687215	66.4619165
467.6391005	324.4743364	62.76168132
462.65538	324.977831	61.48083068
461.82476	324.4743364	62.33473111
456.0104195	324.4743364	61.76546416
464.3166202	323.5232909	62.76168132
460.1635198	325.0897187	62.33473111
464.3166202	325.9288765	62.76168132
455.1797994	330.1246653	62.33473111
460.9941399	330.5162723	62.19241437
469.3003406	329.509283	61.62314742
463.4860001	330.4603284	60.91156373

466.8084804	329.3414514	61.48083068
485.082122	330.0687215	63.75789848
479.2677815	327.1596412	62.90399806
480.0984016	327.0477535	63.0463148
475.1146811	327.2715289	61.19619721
467.6391005	327.6631359	60.48461352
479.2677815	313.34151	61.48083068
470.9615808	346.9078207	61.19619721
459.3328998	352.6140935	59.63071309
432.7530575	352.6700373	80.69359027
492.5577026	352.054655	82.82834134
451.026699	352.9497566	86.24394304
447.7042188	353.8448582	86.1016263
427.769337	345.9008314	80.69359027
427.769337	344.0546843	81.12054048
419.4631363	347.4113153	82.11675765
417.8018962	346.8518768	83.8245585
